# Implementation of COVID-19 Provider Resource Task Force: A provider support initiative during emergency preparedness in a quaternary-care center in western Michigan

**DOI:** 10.1017/ice.2020.1247

**Published:** 2020-10-08

**Authors:** Vetriselvi Moorthy, Mudita Bhugra, Curtis J. Behenna, Manivannan Veerasamy, Michael J. Harrison, Liam R. Sullivan, Russell J. Lampen, Habiba Hassouna, Jorgelina T. de Sanctis

**Affiliations:** 1 Spectrum Health Infectious Diseases/Michigan State University, Grand Rapids, Michigan; 2 Spectrum Health Richard McNamara Cardiovascular Diseases Fellowship/Michigan State University, Grand Rapids, Michigan; 3 Specialties/Digestive Health, Spectrum Health Medical Group, Grand Rapids, Michigan

*To the Editor—*On March 11, 2020, World Health Organization (WHO) announced coronavirus disease 2019 (COVID-19) a pandemic.^1^ Spectrum Health System, like many other healthcare systems, activated an emergency preparedness command center and implemented strategies to mitigate the impact of COVID-19. Spectrum Health System is a nonprofit quaternary-care health system based in Grand Rapids, Michigan. More than 4,600 physicians and advanced-practice providers offer care to patients through 14 hospitals, including Helen DeVos Children’s Hospital and 150 ambulatory clinics. Grand Rapids medical community also includes 2 other health systems, many ambulatory specialty and subspecialty practices, and rehabilitation units. During the COVID-19 pandemic, Spectrum Health initiated a work group as a support initiative for the patient-facing clinicians in the entire community.

## Methods

The COVID-19 Provider Resource Work Group and hotline were commissioned on March 19, 2020. The aims of the work group were (1) to provide real-time provider-to-provider education regarding questions that pertain to COVID-19; (2) to respond to a COVID-19 e-mail inbox that received questions from 31,000 employees within the Spectrum Health System; (3) to enable triage providers to direct their patients to appropriate channels; and (4) to improve provider access to updates on testing, treatment guidelines, and web-based resources. In total, 18 Spectrum Health physicians from infectious disease, allergy and immunology, internal medicine, and pulmonary critical care, as well as infectious disease fellow physicians volunteered to be part of the work group.

Initially, clinical questions from all the providers across the medical community would go through the provider hotline. This hotline was attended by a nurse who directed the appropriate calls to the on-call physician from the work group. Physicians self-assigned themselves to 4-hour shifts 7 days a week from 8 a.m. to 9 p.m. on a secure HIPAA-compliant communication platform called Perfect Serve. A shared inbox “covid19dr” was attached to the physicians’ personal organization e-mail to receive questions. After an initial meeting that was organized to review the goals and objectives, the work group communicated daily to discuss the challenges, work flow, and current literature. Physicians self-educated on the current literature by reviewing Spectrum Health LitCovid, an internally created database maintained by the research department. No additional financial incentive was provided to the physicians; participation was voluntary.

The calls and e-mails were tracked. The daily number of secure messages ranged from 10 to 20 during the first 4 weeks and progressively diminished over the following weeks when providers were more familiar with the process. The daily e-mails numbered 5–10 initially, followed by a steady decrease. Resources and guidelines were updated in real time within the organization website. Physicians documented the questions and the actions taken so they could be discussed at the daily meeting. Calls were escalated to the infectious disease on-call provider if necessary.

### Analysis

The medical community of Grand Rapids, Michigan, like others across the world, was facing uncertainties regarding the novel nature of SARS CoV-2 virus, limited treatment options, shortage of personal protective equipment (PPE), and inadequate testing supplies.^2^ Our provider work group played a tremendous role in the psychosocial well-being of the clinicians by aiding with both personal and professional responsibilities and addressing concerns.^2^

Providers from intensive care unit, inpatient medical units, ambulatory centers, rehabilitation units, hospice unit, outside hospital, and private pediatric and obstetrics offices reached out to the work group. The spectrum of concerns raised included guidance on testing, PPE, isolation precautions, immunocompromised patients, supply chain, and treatment options (Table 1).

**Table 1.** Spectrum of Calls and E-mails Received by the Provider Resource Work Group

Period	Types of Calls and E-mails
Week 1	Use of PPE with negative COVID-19 testing and removing isolation precautions Asthma exacerbation and use of nebulizers with positive COVID-19 test Appropriate N-95 mask use for all providers Appropriate timing to initiate hydroxychloroquine Encephalopathy and fever, is that COVID-19 presenting symptom? Sensitivity of COVID-19 testing Many outpatient offices with question about testing versus self-quarantine at home due to limited testing Oncology offices with questions about COVID-19 testing prior to chemotherapy; recent travel, cough and need for testing; diarrhea in bone marrow transplant patient and COVID-19 testing
Week 2	Help with completion of COVID-19 testing paperwork for the health department Fit testing for N-95 masks Provider unclear on duration of quarantine whether 7 or 14 days Return to work after COVID-19 Patient coughed on providers before getting COVID-19 test, significant exposure or not and enquired the next step No viral swab available for other viral testing, what is the next step?
Week 3	Visitor exception for end of life patient with negative COVID-19 test Routine preoperative testing for COVID-19 Deployment for a retired physician who wishes to volunteer for COVID-19 COVID-19 testing prior to discharge to rehabilitation centers Questions about convalescent plasma infusion and accessibility Queries received about immunomodulator use in elderly patients Private outpatient offices sent queries about referring COVID-19 testing to the Spectrum Health laboratory Can oropharyngeal swab be used for COVID-19 testing? CPAP use in COVID-19 patients
Week 4	Whether operation room is open for elective cases What is the next step if patients or family refuse to wear mask? Guidance on remdesivir and tocilizumab therapy Provider failed N-95 mask fit test Cost and availability of COVID-19 antibody testing for providers COVID-19 patient with clinical improvement but worsening laboratory markers, what is the expected clinical course? Repeat COVID-19 testing positive after 1 month and what is the appropriate time for plasma donation? Families of patients hospitalized for other illnesses requested COVID-19 testing prior to discharge

Note. PPE, personal protective equipment; CPAP, continuous positive airway pressure.

As we gained more experience with COVID-19 patient care and robust testing and we increased availability of PPE and developed protocols, the number of calls and e-mails steadily decreased, indicating that the clinicians rapidly adapted themselves, mastered new skills, and revised practices. With that decrease, meetings were changed to every other day and then were conducted weekly. The work group was concluded 8 weeks later when the providers were more confident with everyday patient care, appropriate testing criteria, and PPE use for COVID-19 patients.

## Discussion

The medical community has come together and responded in a heroic fashion to the challenges posed by the COVID-19 pandemic.^3^ Spectrum Health’s COVID-19 Provider Resource Work Group hotline is one of the many efforts instituted to improve COVID-19 patient care and control the spread in both hospital and ambulatory settings. Concerns from the work group were appropriately streamlined to the respective COVID-19 performance committee. Other quality improvement initiatives were conducted based on these concerns to restructure the work flow and to improve patient care. By making the COVID-19 provider hotline accessible to all providers across the community, Spectrum Health prioritized the professional development of healthcare workers as well as their psychosocial well-being during the pandemic.

Frontline healthcare staff are not only experiencing a rapid increase in the volume and intensity of their work but are also facing additional challenges such as unfamiliar working environments, changing protocols, and unprecedented exposure to COVID-19 with little opportunity for orientation and training.^4^ It is widely recognized that healthcare professionals need evidence-based support initiatives to mitigate the effects of COVID-19 on their current and future well-being.^4^ Spectrum Health’s COVID-19 Provider Resource Work Group is an example of a successful and crucial support initiative in closing the knowledge gap and having a positive impact on the providers, emphasizing the need for support resources during these tumultuous times. Similar support initiatives and resources should be made available for clinicians during any other healthcare emergency or outbreak that has a public health impact.
